# Application of Machine Learning Techniques to Identify Data Reliability and Factors Affecting Outcome After Stroke Using Electronic Administrative Records

**DOI:** 10.3389/fneur.2021.670379

**Published:** 2021-09-27

**Authors:** Santu Rana, Wei Luo, Truyen Tran, Svetha Venkatesh, Paul Talman, Thanh Phan, Dinh Phung, Benjamin Clissold

**Affiliations:** ^1^Applied Artificial Intelligence Institute (A2I2), Deakin University, Geelong, VIC, Australia; ^2^School of Information Technology, Deakin University, Burwood, VIC, Australia; ^3^Neurosciences Department, University Hospital Geelong, Geelong, VIC, Australia; ^4^Stroke and Ageing Research Group, Department of Medicine, Monash University, Melbourne, VIC, Australia; ^5^Department of Science and AI, Monash University, Clayton, VIC, Australia; ^6^Department of Epidemiology and Preventive Medicine, Monash University, Melbourne, VIC, Australia

**Keywords:** electronic records, stroke outcomes, machine learning, discharge destinations, stroke mortality

## Abstract

**Aim:** To use available electronic administrative records to identify data reliability, predict discharge destination, and identify risk factors associated with specific outcomes following hospital admission with stroke, compared to stroke specific clinical factors, using machine learning techniques.

**Method:** The study included 2,531 patients having at least one admission with a confirmed diagnosis of stroke, collected from a regional hospital in Australia within 2009–2013. Using machine learning (penalized regression with Lasso) techniques, patients having their index admission between June 2009 and July 2012 were used to derive predictive models, and patients having their index admission between July 2012 and June 2013 were used for validation. Three different stroke types [intracerebral hemorrhage (ICH), ischemic stroke, transient ischemic attack (TIA)] were considered and five different comparison outcome settings were considered. Our electronic administrative record based predictive model was compared with a predictive model composed of “baseline” clinical features, more specific for stroke, such as age, gender, smoking habits, co-morbidities (high cholesterol, hypertension, atrial fibrillation, and ischemic heart disease), types of imaging done (CT scan, MRI, etc.), and occurrence of in-hospital pneumonia. Risk factors associated with likelihood of negative outcomes were identified.

**Results:** The data was highly reliable at predicting discharge to rehabilitation and all other outcomes vs. death for ICH (AUC 0.85 and 0.825, respectively), all discharge outcomes except home vs. rehabilitation for ischemic stroke, and discharge home vs. others and home vs. rehabilitation for TIA (AUC 0.948 and 0.873, respectively). Electronic health record data appeared to provide improved prediction of outcomes over stroke specific clinical factors from the machine learning models. Common risk factors associated with a negative impact on expected outcomes appeared clinically intuitive, and included older age groups, prior ventilatory support, urinary incontinence, need for imaging, and need for allied health input.

**Conclusion:** Electronic administrative records from this cohort produced reliable outcome prediction and identified clinically appropriate factors negatively impacting most outcome variables following hospital admission with stroke. This presents a means of future identification of modifiable factors associated with patient discharge destination. This may potentially aid in patient selection for certain interventions and aid in better patient and clinician education regarding expected discharge outcomes.

## Introduction

The use of electronic administrative records has become widespread in many settings in recent years. This includes the primary care setting and hospital environment (1). Administrative data in the Australian setting may be in the form of mandatory hospital collected data relating to every hospital episode of care, with the data reported to state health departments, in order to inform health care delivery, resourcing, and financial allocation (2). Administrative datasets include primary and secondary diagnosis codes, coding related to comorbidities, discharge destination, and other demographic data. The ability to harness this data to improve patient care, predict outcomes, and identify risk factors for recurrent disease and readmission means that this has become an important area for research and health metrics (3). The heterogeneity of the data and data systems themselves mean that close collaboration between clinicians and analysts is required. Identifying the type of data available and applying this to appropriate clinical questions not yet answered makes this exciting future area of endeavor. This also increases the importance of accurate data collection. Even more vital is the capture of disease specific factors.

Despite the apparent decrease in stroke incidence, in an aging population, stroke survival, and prevalence is increasing (4, 5). This dramatically increases the societal burden of care. Importantly, stroke outcomes are significantly affected by timely hyperacute therapies such as thrombolysis and endovascular clot retrieval for ischemic stroke (6–8), admission to a specialized stroke unit setting (9), appropriate imaging and secondary prevention therapies (10), dysphagia screening, and early mobilization (11). These interventions directly impact the need for rehabilitation or other discharge outcomes, including the potential need for long-term high-level care, and mortality (12). Understanding the factors contributing to functional outcome after stroke provides a potential target for clinicians to alter their management of patients (13). It is important to clarify if these strategies are routinely implemented through available data and audit processes, which may be best performed by disease specific quality clinical registries (14). Whilst the interventions above are well-proven to influence outcomes and also result in a reduction in hospital length of stay and readmission (15), there may be other novel factors during the admission process that have not been previously captured or studied. Analysis of available administrative data may identify process, structural, and outcome measures not previously recognized.

It is important to acknowledge the limitations of administrative datasets. Functional outcome data for stroke from administrative data may not be well-documented at any stage in the collection process. Stroke severity such as the NIHSS score may not be routinely captured or mandated and is known to directly impact outcomes (12, 15). Standard functional scoring such as the modified Rankin score or Barthel index may not be well-recorded and are not mandated in the electronic data. At best, in some cases, we may only be able to use proxy markers of function, such as in-hospital mortality, or discharge destination. Whilst these surrogate outcomes are well-captured from administrative data, they may not illustrate functional status comprehensively and in particular relation to stroke outcomes, do not inform around the 3- or 12-month clinical status, often used to assess the benefits of interventions in stroke patients. However, the systematic methods used, relatively complete capture of admitted patient data and system wide data collection in administrative datasets make these compelling sources to utilize.

Using machine learning techniques to answer health related questions presents a unique and powerful option for improving diagnosis, treatment, and outcome measures. There are also opportunities for identifying predictive factors impacting patient outcomes. Knowledge regarding patient and other factors associated with certain outcomes may allow future application of measures that influence patient care.

## Aims

We sought to use data from existing electronically collected administrative records to identify risk factors associated with specific outcomes for patients with stroke (both ischemic and hemorrhagic) admitted to a large regional hospital, in Victoria, Australia. In addition, we sought to evaluate the utility of using a large array of available electronic health record data from a cohort of patients, when compared to a cohort of patients with available stroke specific clinical factors, to predict discharge outcomes following hospital admission with stroke, using machine learning techniques.

## Methods

### Study Setting

Barwon Health is a large regional tertiary hospital, located in Geelong, approximately 1 h to the west of Melbourne, the second most populous city in Australia. This health service provides public hospital care to the population of Geelong and surrounding regional areas. The hospital includes a comprehensive neurology service, including acute stroke thrombolysis, dedicated specialized and geographically located stroke unit, and high-level imaging facilities available for acute stroke investigation. The benefits of evaluating this patient cohort include that the majority of patients with stroke are admitted to the public hospital, via the emergency department, rather than local private hospitals. Nearly all cases were likely to be captured for this region as a result. Stroke units in Australia do not currently require formal stroke unit certification, however, designated stroke units are required to adhere to a number of key elements defined in the national stroke services framework (16).

We obtained a comprehensive selection of data fields from the routinely collected electronic administrative data from Barwon Health, for the period 2003–2014. Administrative data refers to both coding and demographic data and is reportable to the state Department of Health and Human Services (2, 17). We analyzed data based on all patients with an admission diagnosis of stroke, using ICD 10 coding nomenclature. Due to the lack of stroke specific data on functional outcomes after the incident event, surrogate outcomes of discharge destination, and in-hospital mortality were thought to be the most appropriate markers of outcome. Comparisons were made between patient admission source i.e., from home, rehabilitation, nursing home, other hospital, and discharge destination, including death in hospital. The comparisons were performed in order of perceived severity of the outcome. Patient admission source is a defined variable collected for all hospital admitted episodes, as opposed to their discharge destination. By ascertaining relevant factors contributing positively or negatively to our defined outcomes, we hoped to be able to understand novel patient, investigation, and management factors associated with our outcomes. Prior ethics approval had been provided for all data use and analysis between Barwon Health and Deakin University in an institutional agreement.

### Dataset

The patient cohort consisted of 2,531 patients with confirmed diagnosis of Stroke or TIA admitted between July 2009 and June 2013. A stroke admission was defined by ICD-10 codes G46, I60-69, G450-453, and G458-459 in the discharge diagnoses (either primary or secondary). For each patient, the index admission was defined as the first stroke admission of the patient starting from 1st January 2009. Patient records available from Barwon Health admissions prior to the index admission were available and were used to construct independent variables. Available data from index admissions and prior admissions included all data reportable to the state Department of Health and Human Services as part of mandatory hospital reporting (2, 17). Our dataset was not able to capture admission data outside of Barwon Health admissions i.e., was not linked to private hospital admissions or admissions to other public institutions. The outcome considered was the discharge destination (home, rehabilitation, or nursing home) if the patient is alive, or death if the patient had died during hospitalization.

### Data Analysis

We considered all available administrative hospital data including static information (age, gender, occupation, insurance types), and time-stamped events associated with emergency visits, hospitalizations, radiological tests, length-of-stay, emergency attendance time, primary and secondary diagnoses, and procedures. The use of cerebral imaging such as CT and MRI in stroke evaluation is an important process measure in helping to accurately diagnose and manage patients and was felt important to include in the analysis. Medication usage data was not available from our dataset. Age was coded as a binary variable (i.e., the age variable or not) in one of 10-year intervals, in line with other stroke community and cohort studies (18, 19). Occupation was a binary of value 1 if it was either pensioner, retired, or home duties and 0 otherwise. Time-stamped events were aggregated over two periods of time prior to the index admission: 0–12 months and beyond 12 months. This resulted in a total of 1,303 features. Models were built to analyse the factors associated with different outcomes [e.g., in-hospital death vs. others (i.e., Discharge to home, Rehabilitation, Nursing home), Discharge to home vs. others] using penalized logistic regression with Lasso (20).

We split the data in time (external validation) with data from July 2009 to June 2012 for derivation of predictive models and July 2012 to June 2013 as validation. Confidence intervals were computed based on 100 bootstrapped derivation cohorts from the original derivation cohorts using sampling with replacement.

Five different comparison settings for each of the three sub-cohorts of stroke [intracerebral hemorrhage (ICH), ischemic stroke, transient ischemic attack (TIA)] are considered, by evaluating factors likely to be associated with the defined outcomes, vs. other outcomes.

- Discharge to home vs. others (rehabilitation, nursing home, in-hospital death) out of all patients
- Discharge to rehabilitation vs. home for patients either discharged to home or rehabilitation
- Discharge to nursing home vs. rehabilitation for patients either discharged at nursing home or rehabilitation
- Discharge to nursing home vs. death
- In hospital death vs. discharge to all other places (home, rehabilitation, nursing home)

Where there were small sample sizes, data were collapsed together for the purposes of comparison.

All data processing was performed off-line using a commercial software package (MATLAB, Statistics Toolbox, The MathWorks Inc., 1994–2014). Prediction accuracy is expressed as the area under the receiver operating characteristic curve (AUC). Missing data were imputed.

Two feature sets were constructed:

1. Features constructed from the electronic administrative record which included all available detailed diagnosis, procedure, and administrative data. This included stroke and TIA related diagnostic codes (I60–I69, G45) relating to primary diagnosis, secondary (comorbidity) diagnostic codes, and all available procedure codes relating to patient admissions. The number of variables was 1,303 (some examples of the types of features included can be seen in the data items listed in the Appendix Figures).
2. Features constructed from more stroke specific clinical data such as age, gender, smoking habits, co-morbidities (hyperlipidaemia, diabetes, hypertension, atrial fibrillation, and ischemic heart disease), types of imaging done (CT scan, MRI, etc.,—an important stroke management process marker), and occurrence of in-hospital pneumonia. Specific stroke risk factors such as alcohol use, anticoagulant use, and obesity are not included in the routine data collection.

## Results

We derived prediction results for three subcohorts of stroke patients (ICH, ischemic stroke, and TIA) in five different settings, as outlined above. All results presented are based on the validation cohort, unless otherwise specified. Patient characteristics and discharge destinations are summarized in Tables 1, 2.

**Table 1:** Patient characteristics.

**No. of patients**	2,531	%
Males	1,346	53.2
Females	1,185	46.8
**Mean age**	72.9 (18.4–99.8)	
<50 years		1.5
50–59 years		9.6
60–69 years		24.5
70–79 years		24.2
80–89 years		32.2
90–99 years		8.0
**Stroke type**		
Transient ischemic attack		25.1
Intracerebral hemorrhage		14.6
Ischemic stroke		37.7
Aneurysm		0.9
Not specified		21.8
**Comorbidities**		
Hypertension		52.5
Atrial fibrillation		15.4
Hyperlipidaemia		6.7
Smoking		13.8
Ischemic heart disease		8.4
**Imaging**		
CT brain		100
X ray chest		91.8
US carotid doppler		43.8
MRI brain		36.8
**Length of stay**	1–5 days	99.1
	>5 days	0.9

**Table 2:** Discharge destination.

**Discharge destinations**	**%**
Home	58.9
Rehabilitation	24.5
Nursing home	5.1
In hospital death	9.4

**Table 3:** Percentage of patients that fit the model in the derivation cohort under five different prediction settings for three sub-cohorts of stroke.

	**Intracerebral hemorrhage**	**Ischemic stroke**	**TIA**
Home vs. others	16.5% (357)	47.1% (830)	87.5% (659)
Home vs. rehab	24.6% (240)	57.8% (677)	93.8% (531)
Rehab vs. nursing home or death (nursing home and death collapsed due to small sample size)	60.7% (298)	65.2% (437)	
Nursing home vs. death	17.8% (117)	35.3% (153)	
Others vs. death	26.9% (357)	11.9% (830)	

*Where there are missing outcomes in the table, this denotes scenarios where derivation is difficult due to the lack of sufficient number of patients. The numbers in parentheses denote total patient numbers in the derivation category for that pair of outcomes*.

The percentage of stroke type found in our cohort is similar to other cohorts. The occurrence of “Not specified” diagnostic codes highlights a key problem in using administrative datasets and is identified as a limitation in other cohort studies (21).

The percentage of patients with specified comorbidities is again similar to other cohort studies (4, 22), although the percentage with IHD was lower. In relation to imaging, 100% of patients underwent imaging with CT scan of the brain, as is standard clinic practice in patients with suspected stroke or TIA, in order to ascertain presence of infarction or hemorrhage, as well as other causes of potential stroke mimics. The majority of patients had a length of stay of between 1 and 5 days, in keeping with findings from local acute stroke audits.

We sought to identify specific predictive factors from our analysis associated with the outcomes we have studied. These factors were items from our administrative data, presented in the figures below as both positively and negatively weighted variables. Table 6 below summarizes factors found to negatively impact the outcome presented. For example, for patients with ICH, patients were less likely to be discharged home vs. to all other discharge destinations (rehabilitation, nursing home, or die in hospital) in older age groups (80–90 years old), had had prior ventilatory support, a history of urinary incontinence, or diagnosis of SAH.

Figures in the Appendix below identify all factors from the administrative dataset that both positively and negatively impact the outcomes being studied and represent weights of the linear model.

## Discussion

Our goal was to compare the utilization of an electronic health record model constructed using a general set of coding data and demographic data, with a model based on a specifically selected set of clinically recognized features, in identifying data reliability, predict discharge destination, and identify risk factors associated with specific outcomes following hospital admission with stroke. Analysis using the electronic health record data provided better prediction of outcome and use of stroke specific factors did not appear to improve the model's reliability. When comparing the data from Tables 4, 5, our data was highly reliable in predicting outcomes in patients with ICH of discharge to rehabilitation vs. nursing home or death, as well as all other discharge outcomes vs. death. In ischemic stroke, the data was reliable at predicting discharge home vs. other outcomes, discharge to rehabilitation vs. nursing home or death, discharge to nursing home vs. death, and all other outcomes vs. death. For TIA, the data proved reliable in predicting discharge home and to home vs. rehabilitation.

**Table 4:** AUC of prediction for three different sub-cohorts of stroke at five different settings.

	**Intracerebral hemorrhage**	**Ischemic stroke**	**TIA**
Home vs. others	0.604 (0.404–0.791)	0.803 (0.746–0.891)	0.948 (0.901–0.955)
Home vs. rehab	0.600 (0.418–0.783)	0.752 (0.683–0.820)	0.873 (0.749–0.996)
Rehab vs. nursing home or death	0.850 (0.737–0.963)	0.818 (0.736–0.801)	
Nursing home vs. death	0.550 (0.245–0.855)	0.902 (0.777–1.00)	
Others vs. death	0.825 (0.698–0.952)	0.881 (0.804–0.959)	

*The features used are constructed from the electronic administrative record. 95% CI for reported AUC is presented in the respective parenthesis. Results with missing values implies invalid CI associated with unstable models, generally resulted from lack of sufficient data*.

**Table 5:** AUC of prediction for three different sub-cohorts of stroke at five different settings.

	**Intracerebral hemorrhage**	**Ischemic stroke**	**TIA**
Home vs. others	0.459 (0.285–0.634)	0.702 (0.634–0.769)	0.794 (0.585–1.00)
Home vs. rehab	0.296 (0.131–0.462)	0.636 (0.558–0.714)	0.729 (0.283–0.996)
Rehab vs. nursing home or death	0.504 (0.346–0.661)	0.767 (0.674–0.860)	
Nursing home vs. death	0.625 (0.369–0.881)	0.778 (0.586–0.970)	
Others vs. death	0.583 (0.424–0.742)	0.808 (0.718–0.899)	

*The features used are stroke specific clinical data. 95% CI for reported AUC is presented in the respective parenthesis. Results with missing values implies invalid CI associated with unstable models, generally resulted from lack of sufficient data*.

**Table 6:** Selected predictive factors associated with the prediction models.

	**Discharge home vs. other outcomes**	**Discharge home vs. to rehabilitation**	**Discharge to rehabilitation vs. nursing home or death**	**Discharge to nursing home vs. death**	**All other discharge outcomes vs. death**
ICH	Older age group (80–90), prior ventilatory support, urinary incontinence, SAH	SAH, prior ventilatory support, prior CT imaging, urinary incontinence, older age group	Prior admission from emergency to the ward, prior CT brain/cervical spine, older age group, prior ventilatory support	Prior ventilatory support, age 70–80, male gender, SAH	Ventilatory support, age >90, prior CT brain/cervical spine, age 80–90, past admission from emergency to ward
Ischemic stroke	Urinary retention, hemiplegia, age group 80–90, allied health input as inpatient, chest X-ray, pneumonitis	Urinary retention, inpatient allied health involvement, hemiplegia, older age group (80–90)	Older age group (>90), pneumonitis, other intestinal disorders, and restlessness/agitation	Other medical care (Z51)^*^, prior ventilatory support, pneumonitis, unspecified threat to breathing, chest X-ray, and hemiplegia	Other medical care, pneumonitis, chest X-ray, and unspecified threat to breathing
TIA	Older age group (>90), cerebral infarction diagnosis, disorientation, prior allied health care and diagnosis of syncope/collapse	Older age group (>90), diagnosis of cerebral infarction, syncope/collapse, and prior allied health involvement	N/A^*^	N/A^*^	N/A^*^

*Factors are those having a negative impact on the outcome in question*.

* *The other medical care (Z51) diagnosis is very broad—includes radiotherapy session, chemotherapy session, blood transfusion without reported diagnosis, preparatory care for subsequent treatment, palliative care, desensitization to allergens, other specified medical care, medical care unspecified*.

There are several problems in using electronic administrative records data to identify risk factors and predict outcomes. The amount of electronic data collection contained in these datasets is copious, and there is significant risk in misinterpreting data if it is not disease specific. The complexities of interactions between patient demographic, diagnostic, imaging, procedural, and outcome data may be difficult to interpret. If there is a small group of well-known risk factors, which have been expertly evaluated or have a sound scientific or peer reviewed connection with the research question or patient group, this may be applied in the analysis. Another method may be to examine a larger group of risk factors and determine their statistical significance and predictive power, and hence refine these to the patient population, using regression methods. However, this method again may not be disease specific. The risk factors used in any analysis may be too limited for the data available, and too much data may make the results noisy or uninterpretable. There are inherent differences in risk factors, measures of severity, and specific management strategies for ischemic stroke/TIA and hemorrhagic stroke, which may be useful to capture in any comprehensive medical record.

The use of logistic regression with Lasso is a common linear classifier method that is also suitable for feature selection. The models obtained are likely to be more parsimonious than logistic regression alone. Our aim was to contribute to understanding about the utility of using electronic health record data for clinical prediction, rather than use of different machine learning methods.

Although we understand risk factors such as age, gender, and co-morbidities well in terms of their likely effect on outcomes in stroke patients, the highly detailed data collected by the hospital data warehouse, both for reporting, planning, and financial purposes, means there are likely to be novel but useful predictive factors identified from analyses like this one. Of interest from our list of identified predictive factors for discharge destination were the findings of prior factors in patient histories including prior ventilatory support, imaging factors, respiratory and urinary tract conditions, and allied health input. These novel past history and other elements may indicate new and innovative areas to focus on, guiding clinically, and patient relevant insights and exploration.

Note that factors for Nursing Home vs. Rehabilitation and Death vs. Others for patients with TIA are not presented since the predictive models are unstable (as seen by the lack of valid data in Table 3).

The burden of stroke is significant, and recurrent events may add significantly to pre-existing disability, with further acute healthcare, career, and economic impact. Being able to better identify factors associated with poorer outcome can help clinicians intensify efforts in certain areas. Predictive measures can be factored into clinical care paradigms in situations where the data is reliable and serve as an additional tool.

Many of the identified factors from the model felt to influence the outcomes in question appear clinically intuitive. Older age group, the need for allied health and complications of illness such as pneumonitis the clinician understands have a substantial impact on good outcomes in patients with stroke and other diseases. However, understanding these specific factors may help us to better define which patients require more attention or intervention, and supports the strength of the dataset. Some of these factors are not modifiable but can help us in prognostication and better informing patients and families.

One of the limitations of this study was the lack of an available functional outcome measure in the electronic data, leading to the use of “surrogate” markers of function on discharge from the acute event. The use of clinically important scores such as the modified Rankin score and NIHSS (23) in most stroke outcome studies is not possible using the current dataset and highlights the important areas of deficit in clinically relevant/disease specific measures from administrative data. The lack of important imaging data such as stroke infarct volume, and stroke specific treatments, is also a barrier.

## Conclusion

The electronic administrative record data for our stroke cohort appeared reliable in outcome prediction for most patients and for different stroke types, when based on discharge destination. Risk factors having a negative impact on the defined discharge destinations provide useful and intuitive patient factors which could allow therapeutic intervention and a clearer understanding of which patients are more likely to have better clinical outcomes following an index stroke. In future, the availability of more stroke specific clinical factors in the dataset, including better clinical outcome variables, will likely aid in improving the validity of our data for analysis and prediction.

## Data Availability Statement

The datasets presented in this article are not readily available because the raw data outputs are no longer available due to changes in University and health service agreements. Requests to access the datasets should be directed to benc73@hotmail.com.

## Author Contributions

All authors contributed to conception and design of the study. SR, WL, TT, DP, and BC organized the database. SR, WL, TT, DP, and SV performed the statistical analysis. BC wrote the first draft of the manuscript. All authors contributed to manuscript revision, read, and approved the submitted version.

## Conflict of Interest

The authors declare that the research was conducted in the absence of any commercial or financial relationships that could be construed as a potential conflict of interest.

## Publisher's Note

All claims expressed in this article are solely those of the authors and do not necessarily represent those of their affiliated organizations, or those of the publisher, the editors and the reviewers. Any product that may be evaluated in this article, or claim that may be made by its manufacturer, is not guaranteed or endorsed by the publisher.

## Figures and Tables

**Figure A1:**
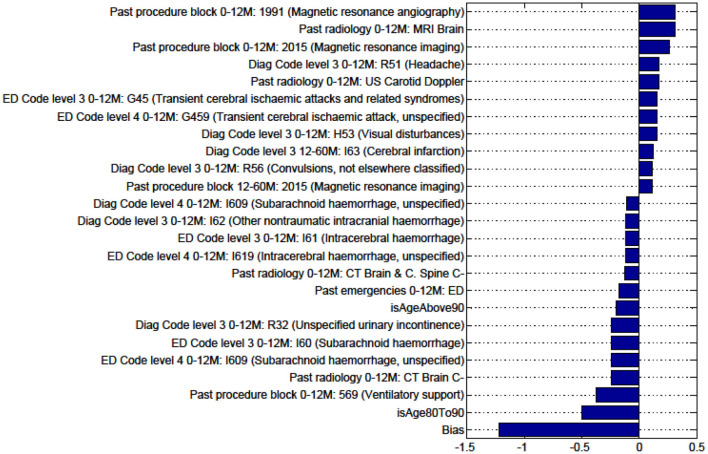
Factors for prediction of Discharge Home vs all Other discharge destinations for the sub-cohort of Intracerebral haemorrhage stroke patients.

**Figure A2:**
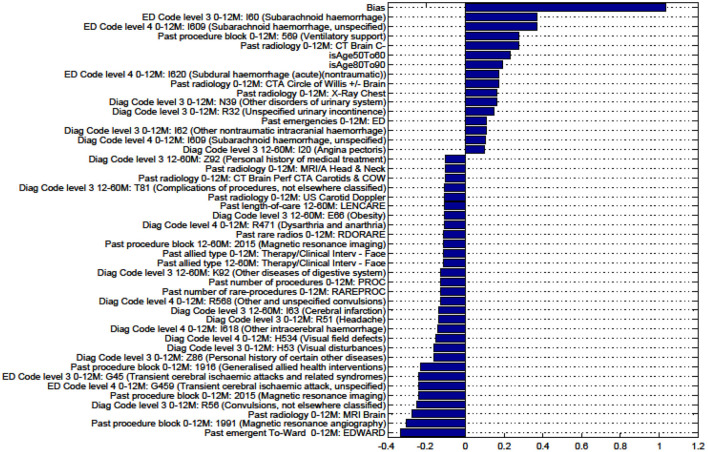
Factors for prediction of Discharge to Rehabilitation vs Home for the sub-cohort of intracerebral hemorrhage stroke patients.

**Figure A3:**
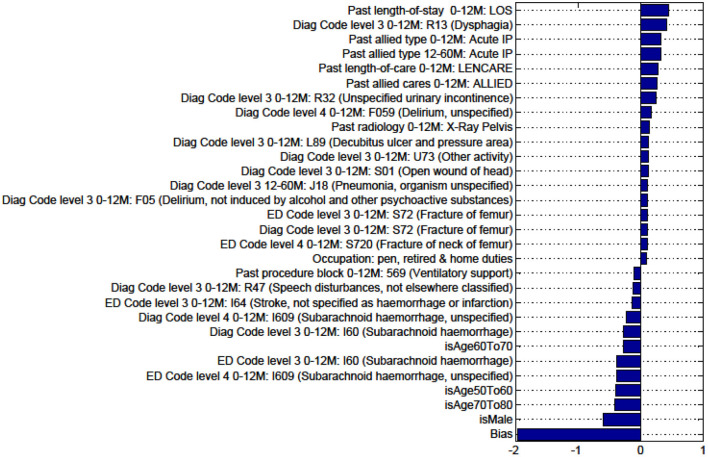
Factors for prediction of Discharge to Nursing Home vs Rehabilitation for the sub-cohort of intracerebral hemorrhage stroke patients.

**Figure A4:**
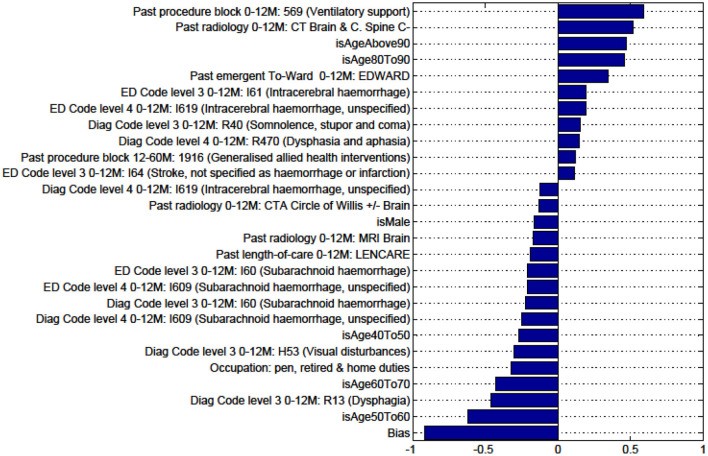
Factors for prediction of Death vs all Other discharge destinations for the sub-cohort of intracerebral hemorrhage stroke patients.

**Figure A5:**
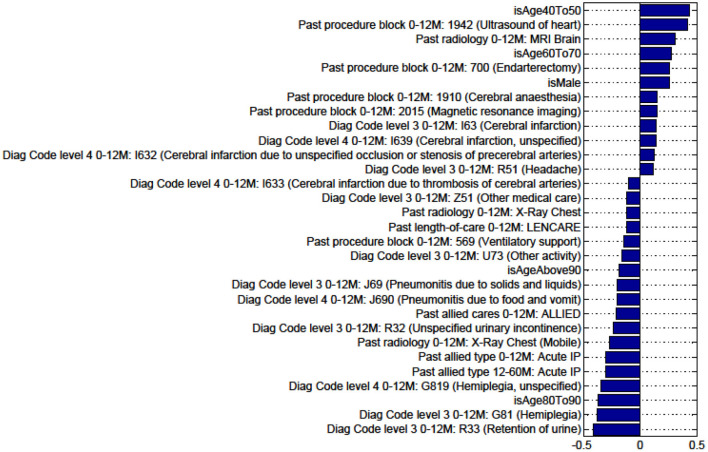
Factors for prediction of Discharge Home vs all Other discharge destinations for the sub-cohort of ischaemic stroke patients.

**Figure A6:**
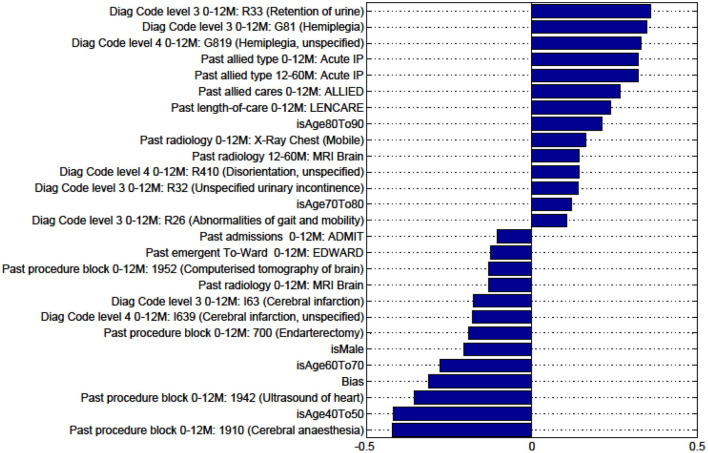
Factors for prediction of Discharge to Rehabilitation vs Home for the sub-cohort of ischemic stroke patients.

**Figure A7:**
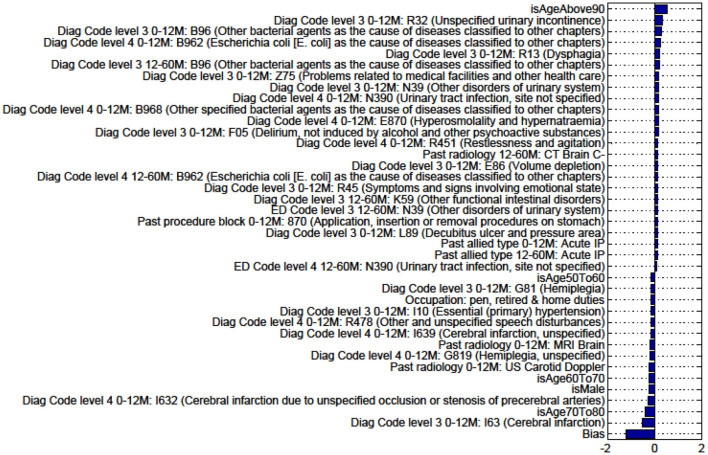
Factors for prediction of Discharge to Nursing Home vs Rehabilitation for the sub-cohort of ischemic stroke patients.

**Figure A8:**
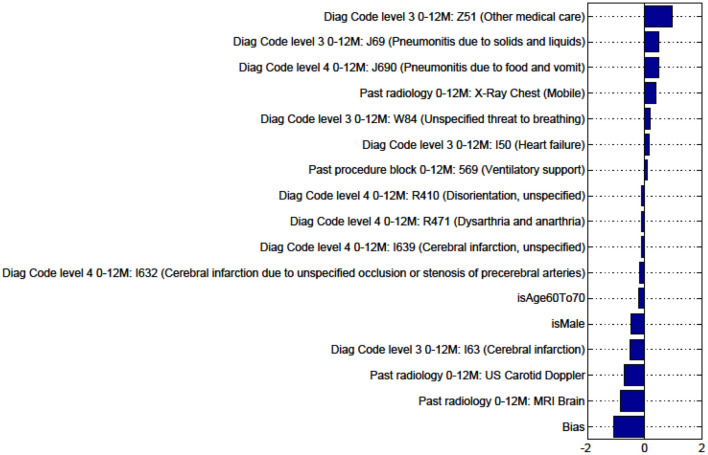
Factors for prediction of Death vs Discharge to all Other discharge destinations for the sub-cohort of ischemic stroke patients.

**Figure A9:**
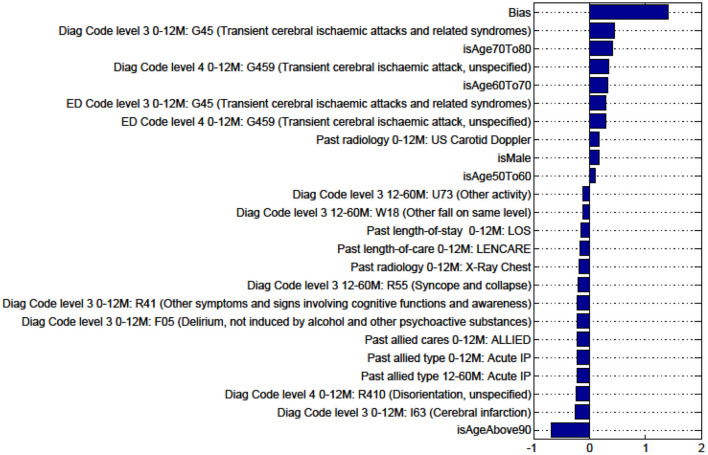
Factors for prediction of Discharge Home vs all Other discharge destinations for the sub-cohort with TIA.

**Figure A10:**
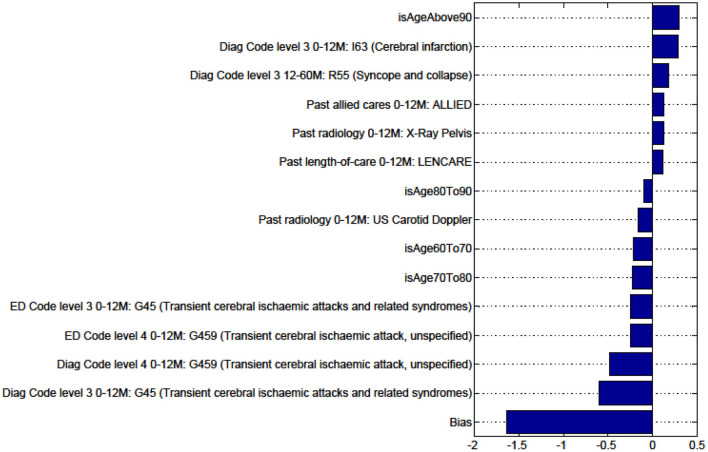
Factors for prediction of Discharge to Rehabilitation vs Home for the sub-cohort with TIA.

## Appendix

Factors for Discharge to Nursing Home vs Rehabilitation and Death vs All Other Discharge Destinations for Patients With TIA Are Not Presented as the Predictive Model Is Unstable.
